# The impact of decentralisation on health systems in fragile and post-conflict countries: a narrative synthesis of six case studies in the Indo-Pacific

**DOI:** 10.1186/s13031-023-00528-7

**Published:** 2023-06-20

**Authors:** Elliot Brennan, Seye Abimbola

**Affiliations:** Sydney School of Public Health Sydney Medical School, University of Sydney, Edward Ford Building A27, Sydney, NSW 2006 Australia

**Keywords:** Health, Health system, Fragile, Conflict, Peacebuilding

## Abstract

A health system has three key stakeholders, the State—at national and subnational levels—the health service providers and the citizens. In most settings and especially in peacetime, these stakeholders are typically well-defined. In contrast, during conflict and crisis as well as during ceasefire and post-conflict peacebuilding, stakeholders in the health system are often more diverse and contested. Health systems in such settings tend to be more decentralised, de facto—often in addition to de jure decentralisation. Despite much debate on the potential benefits of decentralisation, assessing its impact on health system performance remains difficult and its effect is open to dispute in the literature. This narrative synthesis aims to support efforts to assess and make sense of how decentralisation impacts health system performance in fragile and post-conflict countries—by synthesising evidence on the impact of decentralisation on health system performance from six country case studies: Papua New Guinea, the Philippines, Indonesia, Pakistan, Myanmar and Nepal. The impact of decentralisation on health system performance is optimised when combining centralisation (e.g., the benefits of central coordination in improving efficiency) with decentralisation (e.g., the benefits of local decision making in improving equity and resilience). The findings may inform efforts to think through what to centralise or decentralise, the impacts of those choices, and how the impact may change over time as countries go through and emerge from conflict—and as they go through and recover from the Covid-19 pandemic and prepare for future pandemics.

## Introduction

### Background

A health system has three key stakeholders, the State—at national and subnational levels—the health service providers and the citizens [1]. In most settings and especially in peacetime, these stakeholders are relatively well defined through historical precedent and their respective positions of power. While there may be contestation or some fragmentation, they remain relatively stable. In contrast, during conflict, ceasefire, and post-conflict peacebuilding, stakeholders in the health system are often more diverse and contested. Part of successful peacebuilding requires the development or strengthening of institutions to support the functions of state [2]. In fragile and post-conflict states, as elsewhere, the appropriate calibration of health system governance is an important determinant on health system performance. This narrative synthesis aims to support efforts to assess and make sense of how decentralisation impacts health system performance in fragile and post-conflict countries.

### What is health system decentralisation?

A growing body of literature shows that decentralisation can promote quality and access in health systems by improving efficiency, responsiveness, and accountability [3–6]. Since the 1990s, many countries have attempted reforms that look to decentralise aspects of governance. Manor [3] suggests that devolution of power “can ease [ethnic or religious minorities’] alienation from the state and the wider society and reduce the danger of damaging conflict.” Decentralisation represents both a management strategy and the empowerment of local governance structures [4, 7]. Hence, decentralisation has been championed as a strategy to improve governance in fragile states during peacebuilding efforts or transitions to democracy. Various functions of a health system many be decentralised such as decision making, human resources, and financial resources including decisions on allocation. Decentralisation, as a top-down intervention from a central government to lower levels of government, may occur in several ways through *devolution* by a transfer of authority, *delegation* by the transfer of managerial responsibilities, *de-concentration* by the transfer of administrative function, or *privatisation* by the transfer of functions from the public to the private sector [7].

The studied benefits of decentralisation are varied but in high-income countries, fiscal decentralisation has been shown to have a significant positive effect on reducing infant mortality where and only where substantial autonomy in revenue sources has been devolved to local government [8]. Elsewhere, Chol et al. [6] identify health system decentralisation as one of three explanations for a reduction in maternal mortality post-conflict (other explanations were improved government financing and support for community health workers). However, some literature suggests that decentralisation is a relative term defined only by the same country’s historical comparison [9]. Hence, despite much agreement of the potential benefits of decentralisation, its assessment remains difficult and therefore its impact is open to dispute in the literature.

### What are fragile and post-conflict states?

**Fig. 1:**
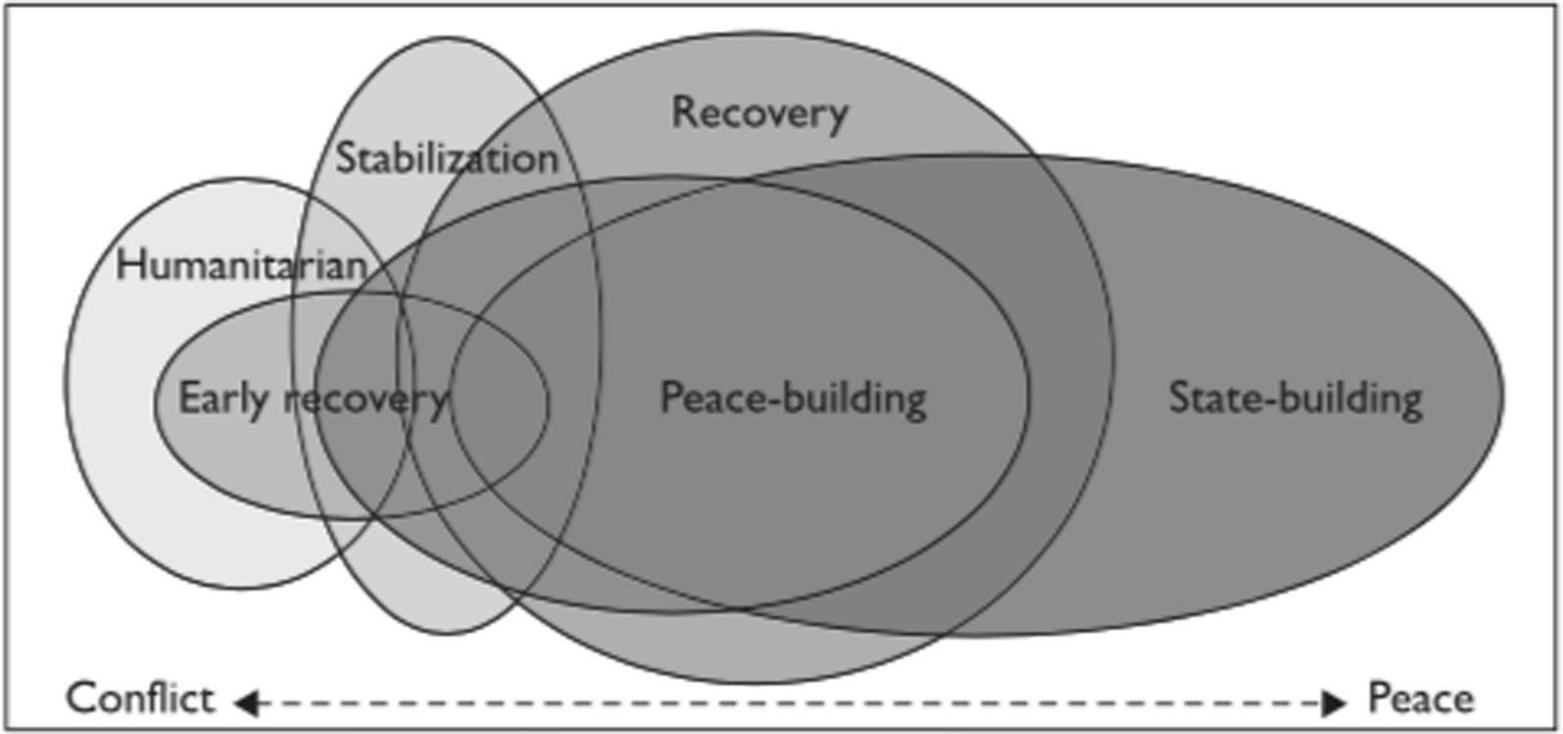
Approaches to conflict and transitional settings, Howard et al. [13]

The term fragile state is heavily debated as it relies on a subjective selection of indicators [10]. One index of fragility, the Fragile States Index, assesses fragility across four indicators (cohesion, economic, political and social) and several sub-indicators to assess individual states. Many of these states have simmering or hot conflicts; therefore, observing the presence of conflict can further support notions of fragility. Peace, like conflict, is not simple or linear in reality. Galtung distinguishes between negative peace, the absence of war, and positive peace the absence of war with the establishment of sustainable peaceful societies [2], which may by extension incorporate health system equity, efficiency, and resilience. During both, to varying degrees, fragility is present. While degrees of fragility may be harder to grasp and more contestable, the absence of hot-conflict, i.e. the absence of fatalities of combatants, is clearer—not the least by the impact conflict has on population health. Health system strengthening in fragile states often focuses on humanitarian relief and not deeper and longer-term systemic improvements [11]. Aid often seeks to stabilise a fragile political situation and respond to immediate population health needs rather than support the longer-term improvement of health outcomes—thus supporting curative rather than preventative health services [12] (Fig. 1).

Armed conflict increases the prevalence of years lost due to ill-health, premature death or disability, as measured by disability-adjusted life years [14]. In addition, more than 70% of disease outbreaks occur in fragile and conflict-affected countries [15] Armed conflict may lead to health infrastructure deteriorating or being destroyed, it may increase displacement and migration and worsen resources, access to and equity of services [16] These combined impacts may lead to more indirect deaths or disability than the casualties from the conflict itself [16]. However, health system governance is often weak before conflict began [17].

The early recovery and stabilisation of a health system are crucial components of building positive peace. A growing body of literature questions the coupling of ending conflict and building democratic institutions through a “liberal peace” [18, 19]. Instead, tying peacebuilding to improved health outcomes may prove less contested and more successful. As Barbara and McQueen [16] note the sub-field of peace through health is largely driven by case studies rather than theory generation—a limitation that this paper seeks to remedy.

Analyses of measures to improve governance (e.g. through decentralisation) in post-conflict states are complicated by the *sui generis* nature of each country [20], including of the internal character of the conflict. Strategies of the international community to support reforms include the central or local provision of aid in the form of money, expertise or resources. But there is also the challenge of coordination of aid and humanitarian assistance in post-conflict environments [7, 21] which may flow to local levels of government where capacity is poor and may weaken overall governance or central government trust in reform programs [7, 21, 22]. It is therefore essential to understand how decentralisation influences health system performance in fragile and post-conflict states.

One study which observed case studies of several fragile states [23] found that the uptake of policy on health system reform was “strongly driven in most settings by local political economic considerations.” Another study found that health system decentralisation was a common link that could explain the reduction of maternal mortality rate [6]. A de facto form of decentralisation also occurs in conflict, where essential services are supplied by local or grassroots level actors [7]. Further complicating reforms is violent resistance to change in such environments—for example, during health system decentralisation in Nicaragua health workers were attacked by the contras—i.e. rebel groups [24].

### What is the Indo-Pacific?

While much of the literature on fragile states examines the Middle East and Africa regions there is less on what is the relatively new geo-political grouping of the Indo-Pacific. The Indo-Pacific, based on the bio-geographic region by the same name, aims to broaden the understanding of modern-day interactions of great powers lying across the landmasses and islands bordering the Indian and Pacific oceans. The countries herein many of which are categorised as fragile (see “Methods” section), make up over half the world’s population, resulting in a large amount of the world’s poor and poor health outcomes. Their borders and governance structures are largely the vestiges of a bygone colonial era and as such they are diverse and often ill-suited to their cultural, geographic or political traditions [25].

Unlike demographics, geographic conditions remain largely unchanged. Climatic conditions have frustrated governance across the Southeast Asia subregion. James C Scott noted that “until recently, state power in Southeast Asia during monsoon season shrank back to the palace walls” [26]. Moreover, to avoid governance, and as some speculate, to avoid epidemics [26], it was common for citizens to flee from population centres to the periphery. Indeed, this was, and in many parts still is, an experience across the Indo-Pacific and crucially informs how de facto decentralisation may occur and how common problems of quality and access may vary temporally and across the health system.

### Objective of the study

While several papers have observed comparative health system decentralisation in Africa [6, 27], particularly in post-conflict settings, the Indo-Pacific, in particular, remains under-studied. Understanding how decentralised governance influences health system performance in post-conflict states within the varied cultural, geographical and socio-economic differences in the Indo-Pacific can inform reform efforts, ultimately supporting more stable and sustainable future for a changing region. Given the over-representation of disease outbreaks in fragile and conflict-affected countries, improving health systems in such countries has wider health security implications. In addition, comparative case studies of health system decentralisation require a theory-generating approach to facilitate the transfer of insights across settings.

As the Covid-19 pandemic has challenged health systems globally, and governments look to strengthen their health systems in response, decentralisation, or centralisation, may be considered as potential strategies. This paper aims to inform where and when decentralisation can improve health system performance, focusing on states emerging from conflict and categorised as fragile.

## Methods

### Search strategy and inclusion criteria

From within this geographical delimitation defined as the Indo-Pacific, countries that had experienced conflict between 1990 and 2016, according to the UCDP/PRIO Armed Conflict Dataset were included for consideration. These included Afghanistan, Bangladesh, China, Cambodia, India, Indonesia, Iran, Iraq, Laos, Malaysia, Myanmar, Nepal, Pakistan, Papua New Guinea, the Philippines, Sri Lanka, and Thailand. To be included, countries also had to be judged by the Fragile States Index 2019 as Warning or Alert countries, i.e. not deemed stable.

Keywords were searched in August 2019 by EB, in consultation with SA, on three databases: Ovid Medline, SCOPUS and Global Health. The selection of the databases followed discussions with colleagues and a librarian at the University of Sydney.

First, a multi-field search of *Global Health* database was conducted using the health system AND conflict AND decentralisation OR decentralization (English-only limit) with a time frame through to 2019. This returned 1259 of which 929 were English results. These were sorted into Indo-Pacific relevant in title, and where this was unclear they were sorted by their Abstract. This database returned 79 potentially relevant papers.

Second, a multi-field search of *Ovid Medline(R)* database was then conducted using health system AND conflict AND decentralisation OR decentralization (English-only limit). This returned 2292 of which 1877 English results. Given the high volume of irrelevant results related to the decentralisation of political system and with low specificity to the health systems, a second search was conducted. In this multi-field search of *Ovid Medline(R)* database, the key words of health system AND decentralisation (English-only limit) were searched with a time frame through to 2019. This returned 81 results of which 76 were English language results. These were sorted into Indo-Pacific relevant in Title, and where this was unclear they were sorted by their Abstract. This database returned 20 potentially relevant papers.

Third, a multi-field search of SCOPUS database was conducted using the keywords conflict AND health AND system AND decentralisation with a time frame through to 2019. This returned 53 results of which 38 were in English. All were included.

Aggregating the results of these databases, and selecting papers that focused specifically on health system reform and decentralisation in a post-conflict or fragile state, that had experienced conflict between 1990 and 2016, according to the UCDP/PRIO Armed Conflict Index, and that appeared in the Fragile States Index 2019 as Warning or Alert countries, there were 27 relevant papers from the following countries: Pakistan (3), Philippines (3), Indonesia (10), Myanmar (2), Nepal (6), Sri Lanka (1), Papua New Guinea (2).

After carefully reading these papers, six countries were selected where relevant literature was strongest. Of these countries were the decentralised archipelagos of Papua New Guinea, Philippines and Indonesia, and the others were the rugged terrains of Pakistan, Myanmar and Nepal. The limited literature on Sri Lanka led to its exclusion from this synthesis.

### Use of grey literature

This synthesis attempts to build upon academic scholarship and draw on lessons from practitioners by including grey literature where relevant, especially government aid effectiveness reports, WHO and think tank reports. Grey literature was found through citations in literature searched as outlined above and through the authors’ prior reading on health system decentralisation.

### Data extraction and analysis

**Fig. 2:**
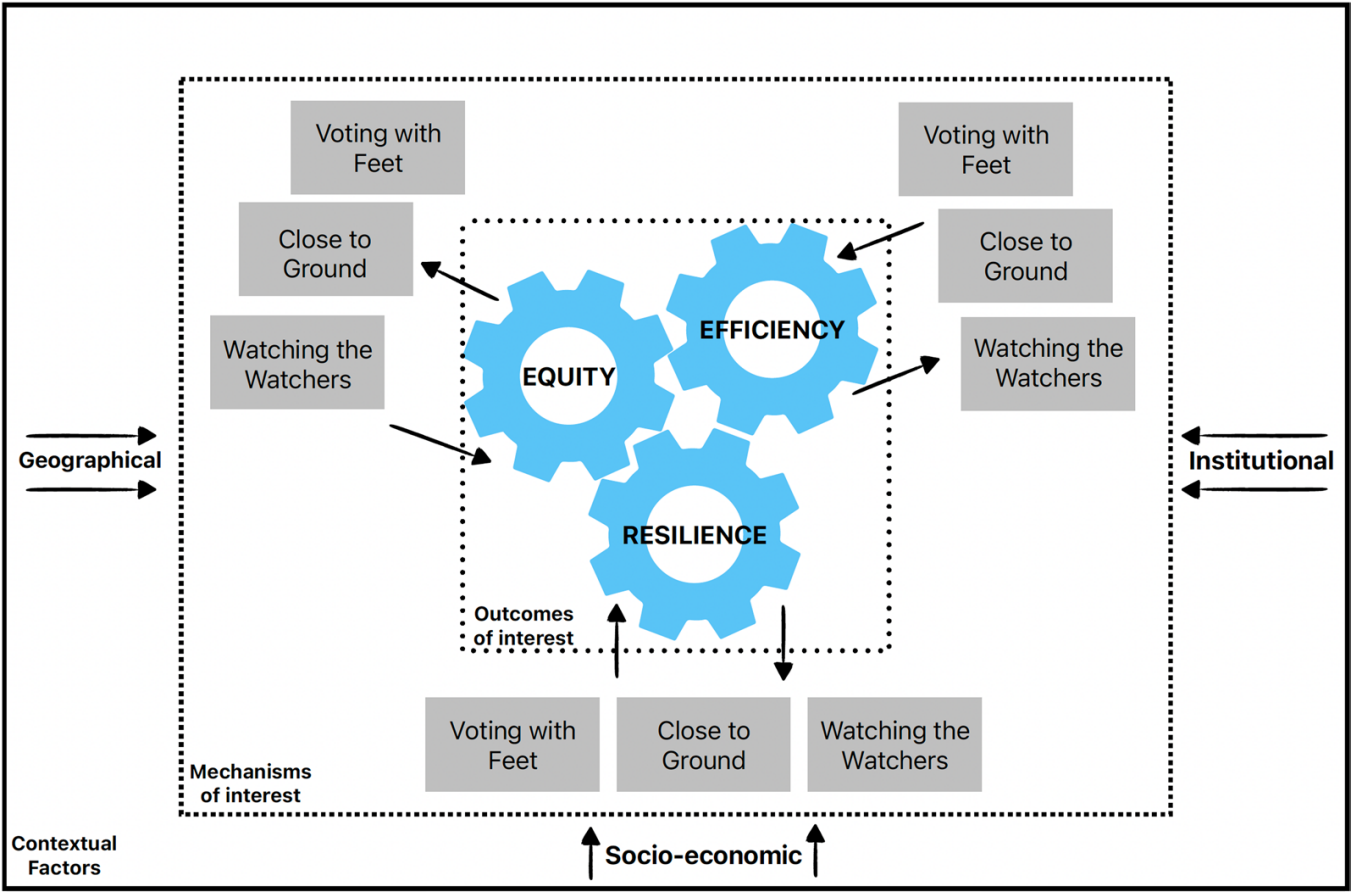
Illustration of the theoretical lens for analysis adapted from Abimbola et al. [29]

This narrative synthesis includes both theoretical discussions of decentralisation and specific case studies. Similarly, we sought to maintain the “vitality, viscerality and vicariism of the…original studies” [28]. We examined experiences of decentralization through health system outcome measures of equity, efficiency and resilience [29]. The analysis was conducted through a theoretical lens put forward by Abimbola et al. [29]. This theoretical lens describes how three mechanisms triggered by decentralisation—‘*voting with feet’*, ‘*close to ground’* and ‘*watching the watchers’*—are influenced by context—*institutional*, *socio-economic* and *geographic context*—to determine the impact of decentralisation on health systems [29]. These mechanisms and contextual factors are described where they appear explicitly in the synthesised literature (Fig. 2).

The narrative synthesis involved three steps, drawing on the realist framework for analysis [30, 31]:

1. Building on existing literature, we identified three outcome categories of interest: Equity, Efficiency and Resilience. In reading the text of each paper, we sought to identify the relevant outcome category. Equity refers to a disparity in health outcomes on the basis of socio, economic, demographic or geographic differences, which would be otherwise avoidable if such differences were resolved [29]. Efficiency refers to the capacity of the health system to respond in an appropriate, timely and cost-effective way to improve health outcomes [29, 32]. Finally, resilience refers to a health system’s ability to respond during and after an emergency, and its overall robustness to acute shock [29, 32].
2. In further exploration of the literature on each country and comparing different accounts and explanations of outcomes across manuscripts, and across countries, we identified three Mechanisms at play in explaining each of the above outcomes of interest: *voting with feet*, *close to ground* and *watching the watchers* [29]. The *voting with feet* mechanism reflects how decentralisation ‘exacerbates or assuages’ existing patterns of inequities in certain jurisdictions relating specifically to individuals, resource allocation or health outcomes [29]. *The close to ground* mechanism reflects how lower levels of governance, acting or deriving policy closer to where outcomes are expected, impacts health outcomes [29]. Finally, the *watching the watchers* mechanism reflects the multiplied relationships of mutual accountability that exist within a decentralised system [29].
3. We identified three sets of contextual factors (socio-economic, geographic, and institutional) which may affect how each mechanism influences each outcome. On further exploration of the identified studies in each country, we identified the contextual factors that may have enabled each of the mechanisms to generate the outcomes that they did, as determined in each of the included studies.

## Results

There were 26 papers from the following countries: Pakistan (3), Philippines (3), Indonesia (10), Myanmar (2), Nepal (6), Papua New Guinea (2). Grey literature was drawn on to challenge and support the papers uncovered in the results from the databases. The Mechanisms at play uncovered in the country-specific literature are explained under each Outcome (Equity, Efficiency, and Resilience) below.

### EQUITY

#### Voting with feet

In Myanmar, equity is impacted by geography, ethnicity and rurality. Higher levels of government retain funds, leaving an imbalance between poorer and wealthier jurisdictions, and between ethnic minority Townships and non-ethnic minority Townships, with consequent poorer health outcomes among people identifying as an ethnic minority and residing in a rural location [7, 33–35]. Regional health departments function where the Myanmar military holds territorial control. In other areas of the country, where ethnic armed organisations hold control, de facto decentralisation by creating separate health services exists with varying degrees of efficacy [7]. In some instances, e.g. in Wa state a region supported by Chinese-state actors [7, 22], services are better than in much of Myanmar and receive medical tourism from Chinese nationals. And for ethnic minorities, *voting with feet* may be more complicated due to racial/ethnic discrimination, prohibitive costs or distances, or may be limited when services are provided by other ethnic health organisations [7, 22, 35, 36].

*Voting with feet* is primarily instructed by existing distribution of wealth [29]. In post-conflict states, sub-national areas that have been in conflict with the state are prone to receive lower health budget distribution and have a greater presence of NGOs that support health services [11]. Health workers in poorer areas may be inclined to move between jurisdictions, such as for higher salaries, better education opportunities or to avoid poverty-related problems. However, for those in minority ethnic areas, access to suitable education and other forms of mobility including finances, appropriate language skills, or familial obligations related to poverty, may limit their ability to move freely [7, 37, 38]. In other cases, a brain drain from rural areas, of competent or qualified individuals moving to higher-paying often urban areas, may lead to reduced capacity in rural areas [39]. Similarly, the local use of an urban health workforce rotating through rural or ethnic minority areas may not achieve optimal health outcomes for rural communities [7].

Shortages in human resources have tangible impacts on policy. The staff shortage in rural areas in Indonesia may have led to an increase in the use of traditional birth attendants [40]. In Pakistan community-based Lady Health Workers provide publicly-funded health services to women in rural areas, helping in part to balance staff shortfalls in rural public health [41]. Health workforce shortages are also impacted by losses to the private sector or international recruitment [42]. Despite human resources continually being cited as a challenge since 2000 in Indonesia, health worker to population ratios either increased only marginally or remained the same [43]. In studies of health workers across 2004–2015, this was a more prevalent problem in the rural areas than urban areas and worse in eastern provinces than in the western provinces, with the worst cases seen in West Papua and Papua provinces [43]. In PNG, local health services, outside of the National Capital District are poor. NGOs and private medical service providers play an important role in rural health service provision, including health services established for international businesses which provide care to local populations outside their employ.

In Indonesia, equity is heavily impacted by the country’s archipelagic geography—the 262 million population are spread over 17,744 islands. Like the Philippines and Myanmar, Indonesia’s health system under both Sukarno and Suharto regimes did not address the country's complexity or diversity of needs [43]. Indonesia’s geography, even with modern transport and communications, has led to a de facto decentralisation of the health system. Since the 1990s, this has been exacerbated in contested areas that have witnessed civil war and armed insurgency such as Aceh, Moluccas islands, Timor Leste and the Papuan provinces. Following the installation of democratic governance, the health system was decentralised in 2001 to 354 districts—today to 514 districts across 34 provinces—which, having increased health system heterogeneity, may have resulted in equity gaps [43]. In an attempt to address this, the UHC system was introduced, covering 203 million people, but with fewer people enrolled from middle wealth quintiles Q2–Q3 and low coverage for infants 0–4 years [43]. A representative from the Western Pacific Regional Office of the WHO described the highly decentralised countries of the Philippines and PNG as their most difficult to support [44], indicating a negative effect on equity of highly decentralised countries, given unequal access to international assistance and support. Similarly, their decentralised nature can be seen as a response to challenging geography with obvious impacts on people’s ability to *vote with their feet.*

Across all case countries, institutional, geography and socio-economic contextual factors loom large. Strong tribal affiliations in PNG, and hostilities toward ethnic groups, as in Mindanao in the Philippines, also impact mobility and may hinder access to health services. Geography however is the major impediment to *voting with feet*. In archipelagic Philippines, PNG and Indonesia geography challenges equitable access to health services. A similar experience is found in people living in highland and rural areas in PNG, Myanmar, Nepal and Pakistan. Where health services remain concentrated in urban centres, rural communities are disadvantaged in access to health services [42]. This is particularly true in PNG and Myanmar where only a fifth and a quarter of the population, respectively, reside in urban environments [7, 42].

#### Close to ground

In Myanmar, Ethnic Health Organisations have recently been formally included in national planning, but health financing remains largely at the central government level with the support of lower levels of governance in development of strategy and laws. However, governing *close to ground* is limited in its ability to contribute towards equity. Even though input is requested from the Township level actors (who also consult with community-level organisations, including EHOs), directives and guidelines still emanate from the central government level [7]. While ethnic armed organisations that run EHOs may be inclined to spend “close to home”, these finances may be reduced or transfers poorly planned to improve health outcomes, thereby impacting equity. Health financing implemented *close to ground* may allow greater local ownership over funding [7, 29]. Funding that is planned in local areas such as by minority ethnic EHOs in Myanmar are more likely to include local considerations, rather than if planned at a central government level that is majority ethnic and has been at war with the minority ethnic groups for decades.

In Pakistan, after significant decentralisation, the private market in health care accounts for three quarters of services [41]. This may negatively affect equity by favouring wealthier and urban populations. Likewise, private health care is, by its design, more accountable to profit than it is to communities and therefore less likely to be swayed by proximity to populations (i.e. less likely to be impacted by a *close to ground* mechanism), except those who can pay. Community-based Lady Health Workers, a female-only cohort numbering 90,000, provide family planning and reproductive health services in rural areas, providing access to services to approximately half the country’s population [41]. This service aims to reach the half of the population who have greater barriers to access to health services due to traditional and systemic gender biases. It does not however address the systemic nature of these inequities. NGOs play a considerable role in the country’s local level provision of health services. Their role may support local access to health services, and alternative health education and information. Zaida et al. [45] found that decentralisation improved politicians’ ownership of health policy and led to resources, planning and innovation improvements.

Experiences elsewhere suggest that decentralization has been implemented in ways that retain central control over decisions [46, 47]. Indonesia’s districts rely on the central government for 90% of resources, with less than a third of public expenditure on health controlled by districts [47]. In 2015, the central government of Indonesia implemented a Health Law mandating 5% of the national budget and 10% of district government budgets to the health sector. Financing has different roles at both the national level and at the provincial and district levels—the National Health Insurance System (NHIS or Jaminan Kesehatan Nasional) funds diagnosis and treatment. In contrast, local governments fund public health (e.g. health promotion and prevention) services [43]. Using decentralisation to implement central rather than locally derived decisions may limit the potential of the *close to ground* mechanism to impact equity.

On the other hand, in Nepal, human resource management at the local level has led to increased local ownership and staff retention [38]. However, the central government is still in control of the financial aspects of health services at the local level [48]. The Philippines Local Government Code of 1991 allows local governments to generate revenue. But the Philippines’ early experience of decentralization may have exacerbated inequities and weakened overall effectiveness of health services [49]. For example, the sensitivity of reproductive health in some communities where faith-based or community pressures are strong, means the decentralization of health services can challenge access and delivery of these services [49]—this is also a concern in Pakistan and Myanmar. As such, equity may be negatively impacted by decentralization.

Civil society organizations may play significant roles in a health system, often in providing health information but at times also health services. This is the case in PNG where churches, receive government subsidies to provide health services [42]. A currently tabled “back-to-basics” reform, which targets rural and primary care, demonstrate ongoing challenges with limited decentralization to date in PNG. Further Provincial Health Authority Framework reforms aim to improve decentralized governance in the country by extending budget and planning capacity to lower levels [42]. These currently proposed reforms could impact on accountability and impact positively on equity. However, governing *close to ground* may come with poorer health outcomes in facilities with, generally, lower capacity, associated with the presence of unfavourable social determinants of health—such as poorer education, economic opportunity, and housing conditions amongst others.

#### Watching the watchers

While Myanmar’s national government has expressed a desire to improve accountability, including through community engagement, it has not specified a role for local health committees at the village or township level. Instead, accountability roles of different levels of government are specified, with civil society organisations “acting as a watchdog with respect to health service planning, delivery and monitoring” [33]. But monitoring and evaluating duties stop at the Township level. *Watching the watchers* is therefore mostly top-down, limiting its potential impact on equity.

In PNG early experiences of decentralisation was such that healthcare workers reported poor professional support and management [50]. More recent “back-to-basics” reforms in PNG attempt to reorient health system reforms. Across the case studies, factionalism, nepotism (patronage networks) and exercises of power at the local level have impeded best practice policy implementation and limited the equity that health system decentralization may bring [38, 51]. Fossati [52] finds that cooperation between different levels of government can have positive local policy impacts, particularly in health insurance.

In Pakistan, reflecting the influence of *institutional* context on equity, the 18th Constitutional Amendment (April 20, 2010) aimed to change a long history of centralized military rule and decentralize power from the president to the prime minister and from the federal government to the provinces. In its original design, it abolished the federal-level Ministry of Health and devolved health to provinces, more precisely under the control of provincial executive and provincial legislature to which the executive is accountable. However, in 2013 a federal-level Ministry of Health was re-established to respond to what was again seen to be a need for central coordination [41].

In PNG, *watching of the watchers* can be seen to occur through close international cooperation between the government of Australia and the central government in an aid program that aims to improve equity and strengthen decentralized service delivery in provinces and districts [42, 53]. In Myanmar, Chinese-state and non-state actor involvement in de facto decentralization of self-administrated zones in northeast Myanmar has replaced or substitutes the central government's role in supporting health service provision [7, 22]. Despite the differences in the nature of engagement, these two examples of foreign actors playing a role, although in the latter case poorly defined, demonstrate the impact of external actors on equity through *watching the watchers*.

Nepal offers an example of equity being supported by *watching the watchers*. A desire for increased accountability was at the root of Nepal’s movement toward health system decentralisation. The Comprehensive Peace Accord’s signed in 2006 led to the elected Constituent Assembly declaring in 2008 a secular federal democratic republic and embarking on a long, deliberative, and successful [54] process to design a new federal constitution. Research at the time of the peace process noted disagreement about health resource allocation, planning and management [55] and that health system decentralisation was positively associated with improved access, utilisation and service delivery [56] Improving primary health care has been set out as a key objective of government [57]. In particular this aims under Article 35 of the 2015 constitution to allow equal access to health care, emphasising *dalit* (marginalised) communities. Gurung and Tuladhar [58] found that in places where local-level community engagement in health facilities was present greater equity (more dalit and women in decision-making processes) was seen, resource access improved and community accountability increased.

### Efficiency

#### Voting with feet

None of the included studies and identified grey literature about the case countries provided evidence as to how “voting with feet” influences efficiency.

#### Close to ground

In Myanmar, the central government currently supports the delivery of primary health care and essential services at the Township level and below. This is notable in its tendency to be efficient [59]. By providing resources and capacity closer to the ground and working through the centrally organised and commanded Township level, the central government prioritises the use of local information in its decision-making through new engagements with Ethnic Health Organisations, local NGOs, and local community-based actors [33]. Township-level actors are now being organised to identify needs and draft plans along national guidelines, while state and regional level actors are working on similar documents at their levels. While such arrangements can facilitate efficiency, the central government retains control. The role of health financing, legislation and national planning remain largely with the central government [7, 33]. As such, the potential of governing *close the ground* to generate efficiency remains constrained.

In Indonesia, unlike in hospitals or health centres, increasing the number of village-led community health clinics (posyandu) per 1000 population has improved the probability of a child receiving full immunisation [60]. A program instituted in 1983 by the Ministry of Health, the posyandu are staffed by a midwife, a nurse assistant, and a vaccinator, as well as *kaders* (unpaid community health volunteers). Nearly 300,000 posyandu are held monthly [43]. However, despite the potential of these local-level initiatives to generate efficiency gains, weak leadership, poor management and poor funding are repeatedly cited as a challenge [40, 43]—all of which may limit the potential efficiency gains from governing *close to the ground*.

In Indonesia, continued low levels of public funding (despite increases) and limited decision-making power at the district level have been suggested as reasons for the poor performance of the health system a decade after decentralisation [51]. District hospitals require government subsidies to operate [51]. Cost recovery rates of service units are mostly less than one as the commercial sections do not generate significant revenue [51]. Directing fiscal allocations to local government alone has not been shown to improve child immunisation rates [60]. It is argued that the capacity and capability of local government is important for achieving efficiency close to the ground [40, 60–63]. Zaida et al. [45] noted that despite increased provincial health allocations, a lack of coordination between provincial and central level in Pakistan impeded effective implementation. Frequent leadership changes at the provincial level [45] may have further impeded this coordination.

#### Watching the watchers

Traditional political culture in Indonesia focuses on a central and authoritarian power structure, with power often power residing in one person such as a sultan [64]. Devolution reforms challenged traditional power relationships [12]. The reforms addressed these traditional norms and practices poorly and may have weakened their effectiveness. One study suggests that knowledge systems and epistemic culture support the maintenance of central decision-making in development planning, explained by dependence on top-down budget prescriptions [61]. Realising the potential efficiency dividends of decentralisation may therefore require understanding and, where appropriate, accommodating existing socio-cultural norms.

Yet such accommodation may require devolution of power to various actors considered at odds with the state. Such problems are present in the Philippines and Myanmar, where over many decades of insurgency, separatists, extremists, communist groups, private armies have supported politicians and been a mainstay in day-to-day governance. This is articulated in the literature on the Philippines, in terms of the appetite for increased “local power” as the “spoils of fiscal decentralisation” have grown larger [65]. Where democratic institutions were weak, decentralisation can lead to elite capture of governance [52]. Lakshminarayanan [49] suggests that these experiences demonstrate that authority should be shared in ways that best improve health outcomes and national health objectives. Similarly, pervasive poverty has supported clientelism as the poor rely on wealthy patrons to survive, with this relationships taking precedence over their relationship with the central government or its institutions [64]. In a survey in Nepal, respondents suggested that decentralisation had increased the vested interests of political parties at the local level [48]. Various forms of elite capture, clientelism and vested interests at local levels, all of which reflect a failure to watch the watchers, therefore limiting efficiency.

Moreover, despite 25 years of devolution of the health services to local levels in the Philippines, Liwanag and Wyss [66] found that decision space for local decision makers was “moderate” or “narrow”. La Vincente et al. [67] suggest that systemic issues have negatively influenced evidence-based planning at the local level in the Philippines. Liwanag and Wyss [66] recommended that the central government improve accountability by better regulating performance at the local level, improving the capacity of local personnel for strategic planning, management and evidence-based policy-making. La Vincente et al. [67] suggest that the central government strengthen its capacity to improve planning processes for the coordination of funding. Elsewhere, like in the nascent reforms in Myanmar, in PNG, provinces make decisions on priorities of the health budget [42]. In Nepal a lack of clarity of leadership on policy—“who does what, who has what” [48]—was cited. Lack of local decision-making power and poor clarity of leadership roles may impede *watching the watchers*, which impedes health system efficiency.

While [9] argue that federal structures work best where political parties are national rather than subnational in character, Fossati [52] notes that decentralisation in Indonesia empowered local government but also tied those authorities to higher levels of government—in effect creating new political patronage networks.

### Resilience

#### Voting with feet

Resilience may be conferred by the inability of people to *vote with their feet* which pushes individuals to support others with similar interests, which may be based on religious or ethnic similarities. The result of this lack of choice to *exit*, as Hirschmann [68] describes it, may lead to greater *loyalty* to the community. In doing so the resilience conferred by community health organisations may be improved. Such experiences are evident in Myanmar [7, 36] where conflict persists. The inability of individuals to *vote with their feet* may have led to similar experiences of community resilience in remote, fragile and conflict areas in southern Philippines, Pakistan, Indonesia, PNG and Nepal.

#### Close to the ground

The character of the armed conflict in Myanmar conflict has brought some elements of governance closer to the ground—from central government to ethnic-population-led government. This is reflected in the emergence of independently operated ethnic health organisations (EHOs) and ongoing attempts to converge them with the national health system [7]. EHOs in Myanmar show how local resilience can lead to de facto decentralisation of health systems. This can be contrasted with how top-down efforts within the framework of decentralised governance have supported resilience in Myanmar, such as through Emergency Operating Centres (EOCs) run by the Ministry of Social Welfare, Relief and Resettlement or Public Health EOCs operated by the Ministry of Health and Sport [69].

Adequate health workforce at the local level is one of the major impediments to functioning of decentralised governance [45, 49, 50]. The organisation of civil society to fill the gaps of a health system show the resilience within communities, such as mentioned in PNG, and demonstrates the capacity for resilience inherent at local levels. The location of services *closer to the ground* may lead to more timely and cost-effective treatment [59]. In effect, governing health services *closer to the ground* increases the decision space of local decision makers and may improve health outcomes and fortify community resilience during health emergencies. Governing close to ground may also facilitate the spread of good practices, improving resilience. There was discussion of subnational jurisdictions in competition, which in Indonesia led to greater policy innovation and thereafter, when successful, imitation by other local administrations [52].

#### Watching the watchers

Where gaps in governance occur, political clientelism increases and non-state actors step in to assuage state weakness. EHOs in Myanmar, where non-state actors are well established and provide health services [7], are examples of *watching the watchers* contributing to resilience. Communities that have been neglected or received insufficient support from the state may be more inclined to seek contributions from outside actors through remittances, foreign actors, aid or trade of illicit goods [7, 70].

De facto decentralization manifests as community resilience when local actors organize to provide community-led health services [7, 22, 70]. This is a product of continued conflict, and other contextual factors that constrain *exit*—such as geography (the areas are largely rural or remote), institutional (discrimination toward ethnic minorities) and socio-economic (relating to opportunity from both rurality and ethnicity). Local governance may also impact resilience, through the ability of local communities to *watch the watchers*, for example, through functioning health boards, whose presence is associated with greater community consultation in the Philippines [71]. Demonstrating the positive feedback loop that happens when local governance functions appropriately, Fossati [52] finds that multi-level cooperation leads to strong policy outcomes even where local democratic institutions are weak.

## Discussion

This narrative synthesis aims to support efforts to assess and make sense of how decentralisation impacts health system performance in fragile and post-conflict countries. The results support conceptualisations in the literature as articulated by Abimbola et al. [29] that optimising the impact of decentralisation of health systems performance requires a careful combination of centralisation, (e.g., the benefits of central coordination in improving efficiency), with elements of decentralisation (e.g., the benefits of local decision making in improving equity and resilience). But this is not without exceptions. There is the example of PNG’s experience of decentralisation, which, having shared central and local authority of provincial health infrastructure, is now being overhauled after years of ineffective operation [42]. Another is reproductive health in the Philippines, where local policymakers under community pressure halted nationally-directed and WHO-supported programs on family planning [49]. These two examples demonstrate that the closer proximity of decision-makers in sensitive policies (*close to ground* mechanism) can also have negative outcomes.

**Fig. 3:**
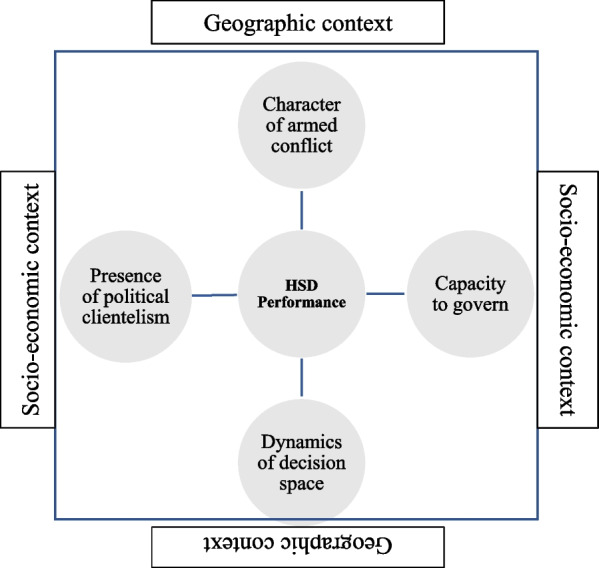
Four factors impacting health system performance under decentralisation

As outlined in Fig. 3, this synthesis identified four institutional factors that may impact health system performance under decentralisation in the observed fragile states. These four institutional factors exist within the context of various socioeconomic and geographic factors.

First, the capacity of local government to function appears to be a common thread in settings where performance is strong or weak. Throughout the literature, a common complaint was insufficient capacity or poor leadership at both the national and local levels. This impacts the three outcomes of equity, efficiency and resilience. As Zaida et al. [45] note, investment in technical capability at both sub-national and national levels may improve health system performance, by improving the implementation of decentralisation reforms.

Second, the dynamics of decision space varied throughout the experiences of decentralisation. For example, policy planning was conducted at a central level with limited local input, transfers were insufficient to local actors or poor coordination or capacity restricted good decision-making. These dynamics have impacted the three outcomes of equity, efficiency and resilience.

Third, the presence of political clientelism was evident throughout the case studies. This was largely blamed on poor governance, coordination or a lack of capacity. Its impacts were felt across equity and efficiency outcomes but may also have influenced some aspects of resilience. Patronage relationships emerged both at the local level and between levels. This may be an intractable issue, not the least within fragile and post-conflict countries where peace, negative or positive, is desired above all else. It is unlikely however that health outcomes of equity, efficiency and resilience will be achieved where negative peace is allowed to persist without movements toward lasting positive peace.

Fourth, the character of armed conflict appears to be important. Where only two conflict actors were present such as in Nepal, successful conflict resolution and health system reforms enshrined in peace accords and legislated appeared to support health system performance under decentralisation. In Myanmar and the Philippines, where multiple actors were present and conflict appears more intractable, de facto decentralisation occurred. In PNG, state fragmentation has led to little improvement in health outcomes and a reversion to a ‘back to basics’ approach has been necessary. This may suggest that beyond the simple number of conflict actors, other factors like the intensity, duration and prospects of victory or defeat are also likely to impact peace processes and resulting health governance.

The various centres of governance created by decentralisation are built around geographical and historical lines. They are subject to old rivalries and power structures, including clientelism and factionalism that impede the capacity to govern and restrict decision space. In active or latent conflict environments state capture by retrenched armed groups, factions or other interest groups is a risk. With a greater number of actors involved in decision-making processes after decentralisation [72], this synthesis suggests that factionalism and clientelism in post-conflict countries may hinder the capacity to govern in decentralised health systems. This is in line with the literature suggesting that in such settings, greater coordination and regulation are needed to enhance efficiency and support democratic institutions at the local level [29, 52, 73].

Unsurprisingly, the mechanisms of *close to the ground, watching the watchers* and *voting with feet* were all evident within the literature with the latter being the least represented. This may be explained by reduced ability to move in fragile and post-conflict environments. By contrast, suspicion of central government following conflict may increase the desire for the mechanisms of *close to ground* and *watching the watchers*.

Throughout the literature, poor planning or insufficient capacity before reforms were suggested to be the cause of poor health system performance [45, 48, 49, 52, 66]. It is important therefore to consider how states arrive at and begin such reforms. As shown in the Samaritan’s Dilemma [74] aid-recipient states can become aid dependent, a likelihood that is potentially worsened in transitioning conflict states [11, 75]. The problem of aid dependency persists in varying degrees in all the countries included in this review. Reliance on aid often means limited resources and capacity before reforms even begin. In turn, limited resources and capacity limit the potential success of decentralisation reforms.

The literature on health system decentralisation synthesised in this review largely omits a potential pathway to progress discussions of the possibility of coupling public health with a liberal-democratic peacebuilding effort—which for example supports civil society organisations and grassroots participation [18]. Instead, the literature explores an alternative potential pathway—i.e. public health serving as a tool for building a resilient society, economic prosperity, and long-term sustainable personal and public growth. This latter pathway to progress, in a post-conflict environment where governance is still geared and often controlled by a centralised power, such as a military or military-appointed government, may prove more effective in fast-tracking the improvement of health outcomes.

More broadly, attempts to achieve “liberal peace”, peacebuilding that attempts to also institute elements of the rule of law, market economy and democracy, have been criticised in the literature as being counter-productive [18, 19]. However, peacebuilding that supports fundamental human security needs has widely been seen as strengthening prospects of peace and a necessary component of positive peace [2]. The reverse has also been noted as true, as initially established in the 1986 Ottawa Charter which recognised peace as a determinant of health [76]. Even then, Fossati’s [52] concerns about the dangers of decentralisation when democratic practices are not institutionalised should be well noted. Both may need to be facilitated hand in hand.

Future studies may look to the wider inclusion of grey literature, and an embedded view, which may be beneficial to deepening understanding of the impact of decentralisation on health system performance, especially in fragile and conflict-affected countries. Policy analysis can observe the policy process or it can embed itself in the process and provide insights into the policy process itself. A hybrid of these two approaches, this study is informed by one of the authors’ (EB) decade-long engagement in policy development and peace initiatives in the Indo-Pacific.

The framing of decentralisation—as either a phenomenon or an intervention—by Abimbola et al. [29] sheds further light on these results. The case studies included in this synthesis have geography-derived features that exemplify the definition of decentralisation as a “phenomenon”. The mountainous geography of Pakistan and Nepal, the rugged and varied terrain of Myanmar, and the archipelagic nature of the Philippines, Indonesia and Papua New Guinea suggest that de jure decentralisation is necessitated by geographically determined de facto decentralisation as many jurisdictions are far from the centre. Such geographically determined decentralisation has to be considered in relation to the implementation of decentralisation as an “intervention”, as decentralisation is also often imposed through a peace process in attempts to stabilise the country and during periods of state-building.

The socio-economic consequences of decentralisation can be similarly found in geographic advantage and disadvantage. For example, Nepal is spread across the mountainous Hilly and Himal regions and lowlands in the Terai region, with 59 recognised ethnic communities and many more linguistic groups [77], the lowland peoples are generally wealthier than the highland people. Sociological theories on the Zomia by William van Schendel and James C. Scott [26] are also helpful in explaining these differences. Some features of the Maoist insurgency in South Asia fit the Zomia thesis of Myanmar and wider Southeast Asia [78], an explanation that helps understand elements of Nepal’s ‘non-state spaces’ and some ethnic people that inhabit them [77]. Indeed, distance from centres of central governance, particularly in archipelagic states, would explain both the motivations for decentralisation and the challenges identified in this narrative synthesis. Together this suggests evidence for framing decentralisation as a phenomenon [29].

Suppose we accept decentralisation as part of a phenomenon that represents cultural and geographic lines of delineation. In that case, it is similarly appropriate to discuss decolonising health system governance so it may find lines of historical best fit. Marchildon and Bossert [9] suggest that Nigeria’s federalism has been relatively successful in part due to its “recognition of geographic domination of distinct ethnic groups.” Similar explanations may be found in successes in case studies herein where limited autonomy, and recognition of geographic domination, allow for increased certainty and more robust health system development. Further study is needed to determine the health implications of such policy.

Rising authoritarianism and changing geopolitical dynamics can lead to state fragmentation in countries of the Indo-Pacific region, as some may seek a return to pre-colonial ethnic-based ‘loosely affiliated localities’, potentially generating even more violent resistance against the state [79]. Decentralisation reforms must therefore consider such potential by paying attention to de facto decentralisation. Moreover, as the region begins to reimagine their health systems and governance in the wake of Covid-19, decentralisation may offer a more responsive and cost-effective health system. Indeed, if adequately tailored and implemented with the right mix of centralisation and decentralisation, such reform can drive the transformations to health equity, efficiency and resilience that help prevent and better respond to future pandemics.

## Data Availability

The datasets used and analysed during the current study are available from the corresponding author upon reasonable request.
